# Delirium Tremens—A New Plan of Treatment*Abstract of paper read before the Southern Medical Association New Orleans, November 10, 1909.

**Published:** 1910-01

**Authors:** Geo. E. Pettey

**Affiliations:** Memphis, Tenn.


For Texas Medical Journal.

### Delirium Tremens—A New Plan of Treatment.*

BY GEO. E. PETTEY, M. D., MEMPHIS, TENN.

This condition is defined as a functional disturbance coming on during the course of chronic alcoholism and is due to accumulation of toxic poison in the blood. These poisons are of both drug and auto-origin. The potency of these poisons is progressively increased by a loss of the fluid element of the blood by excessive perspiration and by difficult absorption of water from the stomach. In fully developed cases the volume of circulating medium is pathologically decreased. The brain is hyperemic in a large majority of cases and enemic in a small per cent. These conditions of the brain is an essential factor in the immediate causation of the delirium. In order to intelligently apply remedies to the control of delirium it is necessary to differentiate the hyperemic from the enemic cases.

The indications in treatment are, support of vital functions, control or arrest of delirium and removal of poison from the blood.

For the purpose of restoring the volume of blood, supporting action of heart and promoting elimination by kidneys, normal salt solution is given by rectum, by hypodermoclysis, and in severe cases intravenously. This is pushed until the entire arterial and venous

---

*Abstract of paper read before the Southern Medical Association New Orleans, November 10, 1909.

systems are filled with fluids to their utmost capacity, then, this fluid is drained off by the bowel with large and repeated doses of Epsom salts, the idea being to practically wash the poison out of the blood by forcing fluids into the system and draining the same off by the bowels and kidneys. Calomel is given in full doses at the beginning of the treatment. Sparteine in doses of two grains is given every two to six hours for the purpose of giving additional support to the heart and promoting action of kidneys. This remedy is classed as our most reliable heart tonic and an efficient, non-irritating diuretic.

The free introduction of normal salt solution gives most reliable support to the heart, dilutes and renders less toxic the poison in the blood, improves the condition of the patient in every respect and does much to allay the delirium, but for the special purpose of combating the delirium, in the hyperemic cases, gelseminine is given in doses of one-twenty-fifth grain every one to two hours until its full physiological effect is developed, unless the delirium and unrest is sooner allayed. This drug is a reliable cerebral sedative and motor depressant and is not incompatible with drug indicated in the hyperemic type of cases, but should not be given in the anemic cases. Strychnia, a drug, the effects of which are directly opposite those of gelseminine, is given for the control of the delirium in the anemic cases. Strychnia is positively contraindicated in the hyperemic cases, but in the enemic case, by increasing the blood supply to the brain it quiets delirium. Alcohol is reduced to a moderate quantity, but not entirely withdrawn during the delirium. Physical restraint is condemned. Opiates and other narcotic and sleep-producing drugs are condemned. They are not only dangerous per se, but interfere fatally with the action of curative remedies.

This plan of treatment has been employed, when indicated, in 450 consecutive cases of chronic alcoholism. Some of these were delirious when admitted, others developed delirium after admission, but in no case did the delirium resist the treatment longer than twenty-fours, and in most cases this symptom was overcome in from six to twelve hours from the beginning of treatment. No death from delirium tremens occurred in the entire series of 450 cases.

---

X-ray shadows of renal calculi very close to the vertebral column should make one suspect stones in one half of a horseshoe kidney.—*American Journal of Surgery.*

